# A single system account of enhanced recognition memory in synaesthesia

**DOI:** 10.3758/s13421-019-01001-8

**Published:** 2020-01-14

**Authors:** Nicolas Rothen, Christopher J. Berry, Anil K. Seth, Sabine Oligschläger, Jamie Ward

**Affiliations:** 1 Sackler Centre for Consciousness Science, University of Sussex, Brighton, UK; 2 School of Psychology, University of Sussex, Brighton, UK; 3 Faculty of Psychology, Swiss Distance University Institute, Brig, Switzerland; 4 School of Psychology, University of Plymouth, Plymouth, UK; 5 School of Engineering and Informatics, University of Sussex, Brighton, UK; 6 Max Planck Research Group Neuroanatomy & Connectivity, Max Planck Institute for Human Cognitive and Brain Sciences, Leipzig, Germany; 7 Faculty of Biosciences, Pharmacy and Psychology University Leipzig, Leipzig, Germany

**Keywords:** Memory, Recognition, Repetition priming, Synaesthesia, Signal detection

## Abstract

Researchers often adjudicate between models of memory according to the models’ ability to explain impaired patterns of performance (e.g., in amnesia). In contrast, evidence from special groups with enhanced memory is very rarely considered. Here, we explored how people with unusual perceptual experiences (synaesthesia) perform on various measures of memory and test how computational models of memory may account for their enhanced performance. We contrasted direct and indirect measures of memory (i.e., recognition memory, repetition priming, and fluency) in grapheme–colour synaesthetes and controls using a continuous identification with recognition (CID-R) paradigm. Synaesthetes outperformed controls on recognition memory and showed a different reaction-time pattern for identification. The data were most parsimoniously accounted for by a single-system computational model of the relationship between recognition and identification. Overall, the findings speak in favour of enhanced processing as an explanation for the memory advantage in synaesthesia. In general, our results show how synaesthesia can be used as an effective tool to study how individual differences in perception affect cognitive functions.

Synaesthesia is a condition in which conscious percept-like experiences are elicited by the presence of a stimulus which does not normally elicit such experiences (J. Ward, 2013). In grapheme–colour synaesthesia, inducers such as numbers, letters, and words involuntarily elicit reliable concurrent colour photisms (Grossenbacher & Lovelace, 2001). Synaesthetes not only have unusual experiences of the world, they also have a distinctive pattern of cognitive abilities. Notably, synaesthetes have enhanced memory performance assessed with direct memory tests which require conscious access to previously presented items (for reviews, see Meier & Rothen, 2013b; Rothen, Meier, & Ward, 2012). However, it is unknown whether synaesthesia affects memory processes which are assessed with indirect memory tests that do not require direct conscious access to previously presented items. Hence, it was our primary aim to test for the relationship between direct and indirect measures of memory in grapheme–colour synaesthesia as compared with yoked controls. Moreover, as a secondary aim, we provide a mechanistic account for our results by applying computational models of direct and indirect measures of memory (Berry, Shanks, Speekenbrink, & Henson, 2012) to further our understanding on the cognitive processes underlying enhanced memory performance in synaesthesia.

Several accounts explain why synaesthesia may be linked to enhanced memory. The first is that synaesthesia enables encoding of additional features (e.g., colour) into the memory representation which may subsequently act as cues at retrieval (e.g., Mills, Innis, Westendorf, Owsianiecki, & McDonald, 2006; Smilek, Dixon, Cudahy, & Merikle, 2002). This has its intellectual roots in the more general dual coding account, in which there is a performance advantage for verbal material due to additional encoding as mental image (cf. Paivio, 1969). However, this account cannot explain why, for instance, visual memory subtests of the Wechsler Memory Scale show at least as strong an effect as in the verbal domain, despite these stimuli not eliciting synaesthesia (Rothen & Meier, 2010; cf. also J. Ward, Hovard, Jones, & Rothen, 2013). This rather suggests that some aspect of memory is atypically sensitive in this group, rather than the alternative possibility that unusual experiences (illusory colours) are grafted on to a typically functioning memory system. Thus, a favourable account is that there are more fundamental differences in the efficiency of some component of the memory system in this group that are not directly tied to the ‘extra’ synaesthetic experiences themselves. However, it remains unclear which specific memory processes may underpin this cognitive advantage. To address this question requires research that contrasts multiple measures of memory.

One candidate mechanism is that (grapheme–colour) synaesthetes have enhanced functioning within the ventral visual stream, but not the dorsal visual stream (Rothen et al., 2012), and, moreover, that these representations support not only perception but also memory (cf. Saksida, 2009). Specifically, the ventral visual stream is related to processes involving high spatial frequency, high contrast, and colour (e.g., words, objects, abstract patterns). By contrast, to the dorsal stream is associated with processes involving low spatial frequency, low contrast, achromatic stimuli, and motion (e.g., spatial perception/memory and attention; Derrington & Lennie, 1984; Kaplan, 1991). Interestingly, synaesthetes more generally tend to think visually (Radvansky, Gibson, & McNerney, 2011; cf. also Meier & Rothen, 2013a) and, for stimuli biasing, ventral visual processing such as high spatial frequency, high contrast, shape and colour, have more finely tuned perceptual discrimination (Banissy et al., 2013; Barnett et al., 2008; J. Ward, Rothen, Chang, & Kanai, 2017). Because graphemes and words consist of high spatial frequency and high contrast information, enhanced processing of these features results in faster access to lexical information. In this view, synaesthesia should also have an impact on indirect measures of memory. So far, the only tests in this domain examined colour-related conditioned responses (Meier & Rothen, 2007; Rothen, Nyffeler, von Wartburg, Müri, & Meier, 2010), artificial grammar learning (Rothen et al., 2013a), and implicit associative learning (Bankieris & Aslin, 2016). However, these studies do not contrast direct and indirect measures of memory. Hence, the present study adopted a previously used continuous identification paradigm with recognition (CID-R; Stark & McClelland, 2000; see also Berry, Shanks, & Henson, 2008a) to contrast recognition memory (direct test) against the indirect measures of repetition priming (henceforth, priming) and fluency.

Recognition memory refers to the ability to judge whether an item has previously been presented in a particular context. Priming denotes a change in reaction time (RT) to identification or production of an item due to prior exposure (e.g., Forster & Davis, 1984). Fluency is used to describe shorter RTs to items judged as old relative to items judged as new, independent of the old/new status (Conroy, Hopkins, & Squire, 2005; Johnston, Dark, & Jacoby, 1985). The CID-R paradigm, which involves separate study and test phases, has the advantage of enabling direct and indirect memory measures to be compared for the same items in the test phase. The study phase consists of masked words that become increasingly visible over time and have to be identified as quickly as possible. The advantage is that the encoding strategy is equated across participants. The same procedure is repeated at test using a mixture of old and new words. Participants are required to identify the word (under speeded conditions) and then report its old/new status (untimed).

It has been demonstrated that a formal single-system (SS) computational model of recognition and priming can account for numerous reported dissociations between recognition and priming (Berry, Kessels, Wester, & Shanks, 2014; Berry et al., 2008a; Berry et al., 2012). A central assumption of the model is that a single memory strength signal drives both direct and indirect measures of memory (with old items having greater values, on average, than new items). Importantly, independent sources of noise are assumed to be involved in the two tasks (noise[recognition] and noise[identification]), and it is the independence in task-specific noise, rather than independence of memory signal, which can account for dissociations. Noise is modelled as a normally distributed variable with a mean of zero. In recognition memory, judgments of old/new are based on whether the value for an item at test (i.e., memory signal + noise[recognition]) is greater or lower than a criterion value. In indirect measures (response times), response times are faster when the strength for an item is greater (i.e., response times are modelled as a decreasing function of the memory signal + noise[identification]). The SS model can be modified to create two multiple-system models (i.e., MS1 and MS2; Berry et al., 2012). The MS1 model is the same as the SS model, with the exception that there are separate and uncorrelated memory strength signals for recognition and priming. The MS2 model lies between the SS and MS1 model. That is, the two separate memory strength signals for recognition and priming may be positively correlated—for instance, via distinctiveness which might lead to better encoding into explicit and implicit memory systems.

Researchers often adjudicate between models of memory according to the models’ ability to explain impaired patterns of performance (e.g., in amnesia). In contrast, evidence from special groups with enhanced memory is very rarely considered. Crucially, these computational models can be adapted to inform the cognitive processes underlying the memory advantage in synaesthesia. The important point is that in the SS model, one continuous variable drives recognition and identification (hence, also priming), whereas in the MS1 and MS2 models, one variable drives recognition and a separate one drives identification. Conscious processing of the inducing stimulus (i.e., the word) is necessary for binding of synaesthetic colour and alphanumeric form (Mattingley, Rich, Yelland, & Bradshaw, 2001; cf. also J. Ward, Jonas, Dienes, & Seth, 2010). Thus, word identification in the CID-R task is predominantly based on lexical information. By contrast, because there is no time constraint on making a recognition decision, synaesthetic colours can be used to promote word recognition (e.g., “I have seen that word because I noticed its colours”). Hence, if the memory advantage in synaesthesia is predominantly based on dual coding, recognition memory will be enhanced, but there will not be an advantage in lexical processing (i.e., word identification and priming). At first glance, a dissociation of this kind could be viewed as evidence against a single-system account in which recognition relies on the same memory signal as priming, and in favour of an account in which a distinct memory signal gives rise to the recognition advantage. Accordingly, we compare the ability of the SS, MS1, and MS2 models to explain the data from synaesthetes.

In line with the notion of enhanced functioning within the ventral visual stream in synaesthesia, we predicted shorter identification times for synaesthetes in comparison with controls. In line with the existing literature and the notion of a perception–memory continuum, we further predicted enhanced recognition memory for synaesthetes in comparison with controls. To gain insight into whether enhanced recognition memory is due to selective enhancement in a recognition signal distinct to that which drives priming, or is due to enhancement of a single underlying memory signal, we compare the ability of the SS, MS1, and MS2 models to the account for the data.

## Method

### Participants

We tested 32 grapheme–colour synaesthetes and 32 nonsynaesthetic controls yoked for age, gender, education, first language, and handedness. In both groups, mean age was 30 years (*SD* = 10 years, range of synaesthetes: 18–57 years, range of controls: 18–55 years), 22 participants were female, 28 were right-handed, and 28 were native English speakers. Synaesthetic experiences were confirmed by testing the consistency of grapheme–colour associations (mean score = 0.75, *SD* = .25) in our sample of synaesthetes (Eagleman, Kagan, Nelson, Sagaram, & Sarma, 2007; Rothen, Seth, Witzel, & Ward, 2013b). On this test, synaesthetes typically score <1 and controls score around 2. None of our controls reported experiencing grapheme–colour associations. Synaesthetes were recruited via our synaesthesia website hosted at the University of Sussex (www.sussex.ac.uk/synaesthesia). Controls were recruited through a University of Sussex participant database and advertisements on notice boards at the university. Participants were tested individually and paid at the rate of £5 per hour for their participation. The study was approved by the local ethics committee of the University of Sussex.

### Materials

A total of 120 four-letter words were selected from the Medical Research Council Psycholinguistic database (Coltheart, 1981). The study phase used 70 words (10 primacy words, 50 midlist words, 10 recency words), and the test phase used 100 words (the 50 midlist ‘old’ items and 50 new items). The two lists of 50 words (Lists A and B) were counterbalanced across each yoked pair of participants, so Lists A and B were used as old/new equally often. The words of List A had a mean frequency of occurrence of 72 (*SD* = 59, range: 10–200; Kucera & Francis, 1967), a mean score of 422 on the imageability scale (*SD* = 54, range: 302–498), and a mean score of 383 on the concreteness scale (*SD* = 62, range: 255–500) in the database. The words of List B had a mean frequency of occurrence of 72 (*SD* = 59, range: 10–200; Kucera & Francis, 1967), a mean score of 423 on the imageability scale (*SD* = 53, range: 307–499) and a mean score of 375 on the concreteness scale (*SD* = 63, range: 244–481) in the database. The remaining 20 words in the primacy and recency trials were in the same range of the specified measures. All words consisted of lowercase letters. Four hash symbols in a row (####) served as mask. Words consisted of black 20-pt Courier font, and the mask consisted of black 26-pt Courier font. All stimuli were presented against a grey background.

### Procedure

**Fig. 1:**
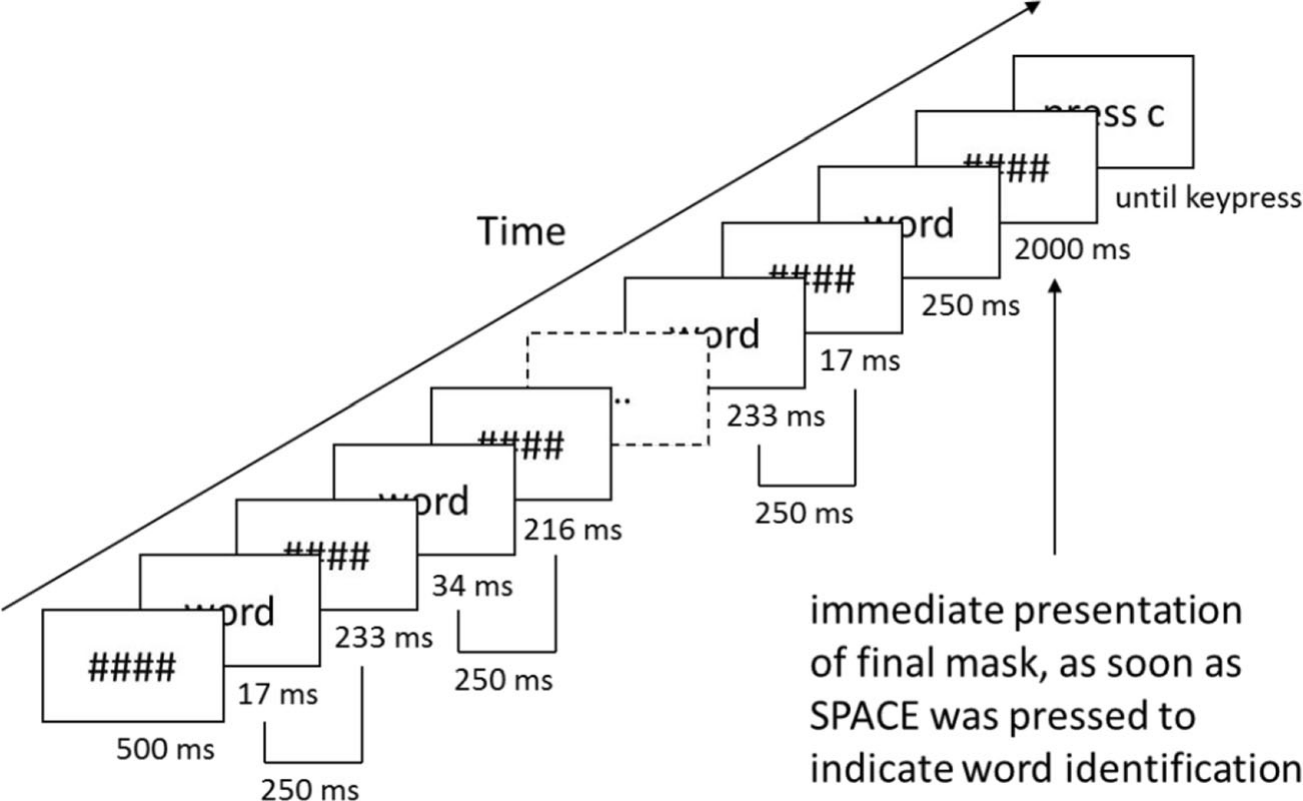
Example trial of the continuous identification task as used in the learning phase (as depicted) and test phase (with additional recognition judgement). During the test phase, after the final mask (i.e., 2,000 ms), participants were asked to make a recognition judgement as to whether a word has been presented during the previous learning phase (i.e., old) or whether it was a new word. The recognition judgement in the test phase was followed by a judgment of the recognition experience (i.e., remember, know, guess)

The experimental procedure was based on Berry et al. (2008a) and consisted of a study and a test phase (see Fig. 1). At the start of the study phase, participants were informed that they would be presented with words flashing on the screen for longer and longer durations, which would make them easier to identify over time. There was no indication of the upcoming test phase. They were instructed to press the space bar on the keyboard as soon as they were able to identify the word, and thereafter to say it aloud. They were advised to do this as fast as possible, but to avoid making errors. Individual trials always started with the presentation of the mask for 500 ms. The initial mask was followed by a 250-ms presentation block consisting of the word displayed for 16.7 ms and the mask for 233.3 ms (the screen refresh rate was set to 60 Hz). This was immediately followed by another 250 ms block, but with the word exposure duration increased by 16.7 ms (resulting in 33.4 ms) and the mask duration decreased by 16.7 ms (resulting in 216.6 ms). The procedure of increasing word exposure duration by 16.7 ms and decreasing mask duration by 16.7 ms was continued until the mask presentation was 0 ms (i.e., 15 blocks in total, or 3,750 ms from the onset of the word after the initial mask, respectively). However, when a response was made during this procedure by pressing the space bar on the keyboard, the mask was immediately presented for 2,000 ms. Below the mask the message ‘Say the word aloud’ was displayed. Thereafter, to start the next trial the instruction ‘Press “C” to continue’ appeared on the screen. RTs were recorded from the onset of a word after the initial mask to the response. RTs longer than 3,750 ms were not registered. In such a case the message ‘Try to be faster on the next trial’ was displayed. Words were presented in random order within their respective list—primacy, midlist, and recency.

The test phase began immediately after the study phase. The general procedure was the same as in the study phase; participants were required to press the space bar on the keyboard as soon as they were able to identify a word. Old and new words were presented in random order. However, after a word was identified, participants were required to judge whether the word was old or new (i.e., one of two designated keys had to be pressed). If a word was judged as old, participants were required to indicate by key press whether they thought it was old because they remembered something specific (remember), it just felt familiar (know), or they were guessing (guess). Similarly, if a word was judged as new, participants had to decide whether they thought it was new because they were sure, it felt unfamiliar, or they were guessing. Thereafter, the instruction ‘Press “C” to continue’ appeared on the screen to start the next trial.

Next, synaesthetes, but not controls, were presented again with all the words from the test phase, one at a time, in random order. Each word was accompanied, on the same screen, by a palette of 13 basic colours, the same each time, but randomly arranged on each trial. Participants were required to select the colour which best matched the colour elicited by the word. If a word did not elicit a colour, they were asked to choose black (for a similar method, see Rothen & Meier, 2010).

### Analysis

The data reported here are available at the Open Science Framework (https://osf.io/nyqbp/ or doi:10.17605/OSF.IO/NYQBP). For the analysis of the study phase, primacy and recency trials were not taken into consideration. All trials in the study and test phase which elapsed without key press, trials with delayed key press (where the word was articulated before the key press), misidentification trials, and trials with RTs less than 200 ms were regarded as errors and excluded from the analysis. Only trials that were correct in both phases in this respect entered the analysis of the test phase. The alpha level was set to .05 for all statistical analyses, and *t* tests were two-tailed. We applied the Greenhouse–Geisser correction where the assumption of sphericity was violated on tests involving repeated-measures factors with more than two levels.

### Computational models

Full details of the models which fit both RT and responses can be found in previous articles (Berry et al., 2012; Berry et al., 2014). The SS model is based on signal detection theory (Green & Swets, 1966); a core assumption is that each item at test is associated with a memory strength variable, *f*, which is a normally distributed, random variable, with mean μ and standard deviation σ_*f*_ (i.e., *f* ~ *N*(μ, σ_*f*_)). Because of exposure during the study phase, the mean *f* of old items is assumed to be greater than that of new items (μ_old_ > μ_new_). To generate a recognition judgment for an item, its value of *f* is first added to *e*_r_ to give *J*_r_, where *e*_r_ is an independent, normally distributed random variable with a mean fixed to zero and standard deviation of σ_r_—that is, *J*_r_ = *f* + *e*_r_, where *e*_r_ ~ *N*(0, σ_r_), and *e*_r_ represents noise that is specific to the recognition task. As in signal detection theory, if an item’s value of *J*_r_ exceeds a criterion, *C*, it will be judged old, or else it will be judged new. For a given item, the same value of *f* that was used to generate *J*_r_ is also used to generate its identification RT in a CID-R task. An important difference, however, is that *f* is subjected to another independent source of noise, *e*_p_, and the identification RT is assumed to be a decreasing function of *f*—that is, RT = *b* − *sf* + *e*_p_, where *e*_p_ is a normally distributed random variable with a mean fixed to zero and a standard deviation of σ_p_ (i.e., *e*_p_ ~ *N*(0, σ_p_)), and *b* and *s* are scaling parameters, which represent the RT intercept and slope, respectively. Thus, the greater the value of *f* of an item, the more likely it is to be judged old, and the more likely it is to have a relatively short identification RT. Old items are therefore more likely to be judged old than new items and show a priming effect. Furthermore, because σ_p_ is typically greater than σ_r_, as μ_old_ increases, this will tend to have a larger effect on recognition than priming. The model represents the idea that the word recognition advantage in synaesthesia is driven by the same signal as word identification, and is not based on a second independent signal.

Under a dual coding account, colour information would be a factor that affects recognition and not priming, and this, in principle, should weaken the association between the two. It seems reasonable to ask whether colour information is simply a factor that affects the recognition noise parameter *e*_r_, and, if so, whether changing its standard deviation σ_r_ would enable the SS model to capture the effects of dual coding. Although it is true that increasing σ_r_ would weaken the association between identification RTs and recognition decisions (all other parameters being held constant), this change would result in a lower predicted value of *d*′, and so the model would not simultaneously be able to predict the recognition enhancement in synaesthesia. Thus, the effects of dual coding cannot be captured by the *e*_r_ parameter.

The MS1 and MS2 models are modifications of the SS model. The MS1 model is the same as the SS model but includes a distinct memory strength signal for the ‘explicit’ (i.e., *f*_r_ drives recognition) and ‘implicit’ (i.e., *f*_p_ drives priming) parts of the memory task, and *f*_r_ and *f*_p_ are used analogously to *f* in the SS model to model *J*_r_ and RT, respectively. In the MS1 model, *f*_r_ ~ *N*(μ_r_, σ_*f*_) and *f*_p_ ~ *N*(μ_p_, σ_*f*_), where μ_r_ and μ_p_ are free parameters, and *f*_r_ and *f*_p_ are uncorrelated (i.e., *r*(*f*_r_, *f*_p_) = 0). This allows the MS1 model to produce independent effects of a variable upon recognition and priming and also conditional independence of the RT and judgment. As such, the idea that the advantage in word recognition memory in synaesthesia is based on a signal, independent of that which drives priming, is directly represented in this model.

The MS2 model is a weaker representation of the notion that colour information is driving the recognition advantage in synaesthesia (i.e., a ‘weaker’ version of the MS1 model). The model is identical to the MS1 model, except that explicit and implicit memory strength signals can be positively correlated (i.e., *r*(*f*_r_, *f*_p_) ≥ 0), for example, due to distinctiveness (with correlation *w*). That is, increased distinctiveness may increase encoding efficiency for both colour and word information. The MS2 model can produce any result that the other models, SS and MS1, can. Keeping average memory signal strength μ_r_ and μ_p_ equal and setting *w* to 1, the model reduces to the SS model. Allowing μ_r_ and μ_p_ to vary independently of one another and setting *w* to 0, the model reduces to the MS1 model (cf. Berry et al., 2012).

### Model fitting

The SS, MS1, and MS2 models were fit to the data using maximum likelihood estimation (see Berry et al., 2014; Berry et al., 2012). A likelihood value can be obtained for every trial in the test phase, given particular parameter values. An automated search procedure was used to find the parameter values that maximized the summed log likelihood across trials. As in previous applications of the models (e.g., Berry et al., 2014), there were five free parameters in the SS model: μ, the mean *f* of old items; σ_p_, the standard deviation of the noise associated with RT generation (*e*_p_); *b*, the RT intercept; *s*, the RT scaling parameter; and *C*, the decision criterion. The MS1 model has five free parameters: *b*, σ_p_*,* and *C*, as in the SS model, and also μ_r_ and μ_p_, the mean of the explicit and implicit item strengths, respectively. Finally, the MS2 model contained six free parameters: In addition to the five free parameters of the MS1 model, the parameter *w*, representing the correlation between *f*_r_ and *f*_p_, was free to vary*.* As in previous studies, numerous parameter values were fixed: The mean of *e*_r_ and *e*_p_, the noise variables for recognition and priming were set to equal zero; σ_*f*_, the standard deviation of *f* (in the SS model) and *f*_r_ and *f*_p_ (in the MS1 and MS2 models), was set to equal √0.5; σ_r_, the standard deviation of the recognition noise (*e*_r_) was set to equal σ_*f*_; the mean *f* (in the SS model) and mean *f*_r_ and *f*_p_ (in the MS1 and MS2 models) of new items was fixed to zero; and finally, the value of *s* in the MS1 and MS2 models was fixed to the estimate of *s* in the SS model. Separate models were fit to the data from each individual, giving one set of parameter values per participant. The Akaike information criterion (AIC) and Bayesian information criterion (BIC) were calculated for each model. The AIC and BIC are measures of the goodness of fit of the model that take into account the number of free parameters (model complexity); lower values indicate better complexity/fit trade-off.

## Results

### Word identification at study

The number of errors was on average 4.6% (*SE* = 1.1) for the synaesthetes and 3.5% (*SE* = 1.0) for the controls. Therefore, no further analysis of the errors was conducted. Mean RTs for word identification were 1,373 ms (*SE* = 46) for the synaesthetes and 1,433 ms (*SE* = 58) for the controls. They did not significantly differ from each other, *t*(62) = .82, *p* = .417, Cohen’s *d* = .20.^1^

### Word identification at test

**Fig. 2:**
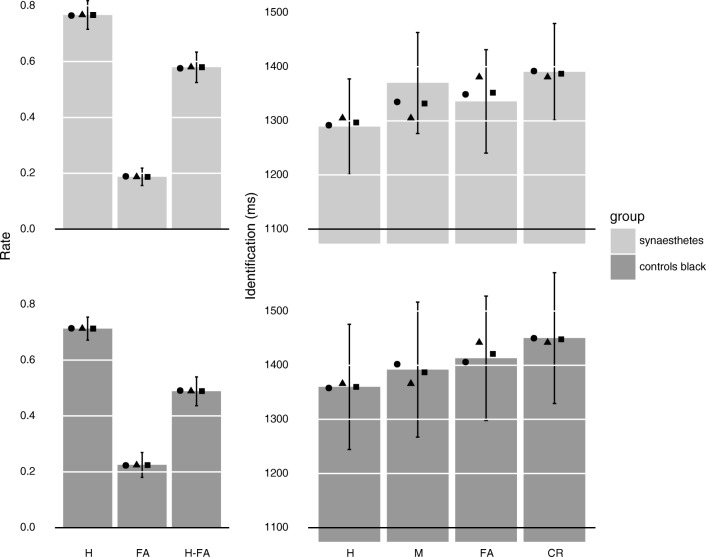
The upper panel represents the data of the synaesthete sample (light-grey bars). The lower panel represents the data of the control sample (dark-grey bars) who were presented with black words. Left-hand side: Bars represent hit and false-alarm rates for the recognition task of the synaesthete and control sample, respectively. Right-hand side: Bars represent RTs for the continuous identification task in milliseconds to hits, misses, false alarms, and correct rejections of the synaesthete and control sample, respectively. Error bars represent 95% confidence intervals of the empirical data. Black dots represent the mean expected SS model results across participants, triangles the MS1 model results, and squares the MS2 model results

The number of excluded trials was on average 3.5% (*SE* = 0.9) for the synaesthetes and 3.2% (*SE* = 0.7) for the controls. Therefore, no further analysis of the errors was conducted. Mean RTs for word identification were 1,343 ms (*SE* = 45) for the synaesthetes and 1,404 ms (*SE* = 61) for the controls. They did not significantly differ from each other, *t*(62) = .81, *p* = .421, Cohen’s *d* = .20. RTs to hits, misses, false alarms, and correct rejections are depicted in Fig. 2 and will be addressed in the priming and fluency section after the presentation of recognition performance. There were no participants with zero responses in the different response categories (hits, misses, false alarms, and correct rejections). Thus, if not further specified, each analysis includes all participants.

### Recognition memory

Recognition performance was measured as *d′*. For the synaesthetes, the average *d′* was 1.75 (*SE* = 0.10; mean hit rate = 0.77, *SE* = 0.03; mean false-alarm rate = 0.19, *SE* = .02, cf. Fig. 2 Recognition) and for the controls was 1.43 (*SE* = 0.09; mean hit rate = 0.71, *SE* = 0.02; mean false-alarm rate = 0.22, *SE* = 0.02, cf. Fig. 2 Recognition). Both groups performed above chance, *t*s(31) > 16.24, *p*s < .001, Cohen’s *d*s > 4.05, and the synaesthetes outperformed the controls *t*(62) = 2.36, *p* = .021, Cohen’s *d* = .59. Response bias, as measured by criterion c (calculated as c = −0.5(z(H) + z(F)), where H denotes the hit rate and F denotes the false-alarm rate), was on average 0.08 (*SE* = 0.06) for the synaesthetes and 0.11 (*SE* = 0.06) for the controls. For the synaesthetes, c was not significantly different from zero, *t*(31) = 1.31, *p* = .201, Cohen’s *d* = .20. For the controls, there was a trend for c to differ from zero, *t*(31) = 1.86, *p* = .073, Cohen’s *d* = .46. However, c did not significantly differ between the two groups, *t*(62) = .41, *p* = .680, Cohen’s *d* = .10. Also, alternative measures of response bias such a beta, log(beta), or normalized c′ did not significantly differ between the two groups, all *t*s(62) < .65, all *p*s > .521, all Cohen’s *d*s < .17.

### Priming

Mean RTs to hits, misses, false alarms, and correct rejections for synaesthetes and controls are depicted in Fig. 2 (Identification). Priming was calculated on an individual basis as the mean RT for new items (i.e., false alarms, correct rejections) minus the mean RT of old items (i.e., hits, misses). Priming was greater than zero for the synaesthetes, *t*(31) = 10.21, *p* < .001, Cohen’s *d* = 2.55 (*M* = 76 ms, *SE* = 7) and the controls, *t*(31) = 6.77, *p* < .001, Cohen’s *d* = 1.69 (*M* = 74 ms, *SE* = 11). The groups did not significantly differ from each other, *t*(62) = .15, *p* = .877, Cohen’s *d* = .04. Consistent with previous research with nonsynaesthete controls (Berry et al., 2008a; Stark & McClelland, 2000), neither of the groups showed significant correlations between priming and *d′* (recognition accuracy), *r*s(32) < .26, *p*s > .173.

### Fluency

Fluency was calculated on an individual basis as the mean RT for items judged as new (i.e., correct rejections, misses) minus the mean RT for items judged as old (i.e., hits, false alarms). Fluency was greater than zero for the synaesthetes, *t*(31) = 9.83, *p* < .001, Cohen’s *d* = 2.46 (*M* = 88 ms, *SE* = 9) and the controls, *t*(31) = 5.07, *p* < .001, Cohen’s *d* = 1.27 (*M* = 61 ms, *SE* = 12). Fluency did not significantly differ between the synaesthetes and controls but there was a trend for synaesthetes to show greater fluency, *t*(62) = 1.8, *p* = .077, Cohen’s *d* = .45.

### Modelling

We sought to explore the potential of the models to explain the pattern of recognition and priming in our sample of synaesthetes and controls. The SS model provided better (i.e., lower) AIC and BIC values than the MS1 and MS2 models for both groups (see Table 1). For both groups, the fit of the SS model was substantially better than the MS1 model (differences in AIC and BIC >8) and was also better than the MS2 model (differences in AIC >33 and BIC >239). These results suggest that the recognition and priming data of both groups are more parsimoniously explained as being driven by a single underlying memory strength signal, rather than distinct memory signals. Model recovery simulations, assessing the ability of each model to be identified by the AIC and BIC had it truly generated the data, did not alter this conclusion (see the Appendix). The expected model results are shown in Fig. 2. It is evident that the SS model closely reproduces the trends in both groups (Table 2).

**Table 1:** Goodness of fit of the models

		Synaesthete group (*N* = 32)			Control group (*N* = 32)		
Model	*p*	ln(L)	AIC	BIC	ln(L)	AIC	BIC
SS	5	−23,071.9	**46,463.7**	**47,431.6**	−23,354.1	**47,028.1**	**47,996.3**
MS1	5	−23,088.7	46,497.5	47,465.4	−23,358.2	47,036.4	48,004.6
MS2	6	−23,065.6	46,515.1	47,676.5	−23,345.2	47,074.4	48,236.2

*Note.* L = maximum likelihood (summed over trials); AIC = Akaike information criterion (Akaike, 1973), calculated as AIC = −2ln(L) + 2Np, where p is the number of free parameters for each model, and N is the number of participants; BIC = Bayesian information criterion (Schwarz, 1978), calculated as BIC = −2ln(L) + Npln(q), where q is the number of observations; q(synaesthetes) = 3,131, q(controls) = 3,137. Smaller AIC or BIC values indicate superior complexity/fit trade-off. BOLDFACE indicates the model that fit the data best according to the AIC or BIC measure

**Table 2:** Mean estimated parameter values of the models

	SS		MS1		MS2	
	SYN	CON	SYN	CON	SYN	CON
Parameter						
μ_r|old_	1.74	1.44	1.75	1.43	1.76	1.43
	(0.58)	(0.48)	(0.58)	(0.50)	(0.58)	(0.50)
μ_p|old_	= μ_r|old_	= μ_r|old_	1.70	1.57	1.70	1.58
			(0.71)	(0.71)	(0.71)	(0.70)
*w*	= 1.00	= 1.00	= 0.00	= 0.00	0.76	0.54
					(0.42)	(0.43)
*C*	0.95	0.83	0.95	0.83	0.95	0.83
	(0.39)	(0.45)	(0.39)	(0.45)	(0.39)	(0.45)
*b*	1384	1439	1381	1442	1381	1442
	(259)	(349)	(260)	(347)	(260)	(347)
*s*	49	51	= SS	= SS	= SS	= SS
	(23)	(38)				
σ_p_	243	254	244	253	242	253
	(52)	(76)	(52)	(76)	(52)	(75)

*Note.* A value preceded by an equal sign indicates that the value was fixed. Standard deviations are shown in parentheses. See the text for details of other fixed parameters. SYN = synaesthetes; CON = controls

To supplement these modelling results, we conducted model recovery simulations; these assessed the ability of each model to be identified by the AIC and BIC, had it truly generated the data (see the Appendix). The simulations indicated that both the SS and MS1 models could be successfully recovered. The MS2 model, however, could not be recovered, and tended to be mimicked by the SS model, and also, to a lesser extent, the MS1 model. The MS2 model could not be recovered because the penalty it pays when the AIC or BIC is calculated is greater than the improvement in fit that its additional free parameter provides. This means that although the AIC and BIC values provide strong evidence against the MS1 model, they do not provide strong evidence against the MS2 model. Nevertheless, we maintain that the SS account of the data should be preferred over that of the MS2 model on the basis of parsimony, because the SS model explains the data with a single strength signal, rather than multiple signals.

## Discussion

The main objective of our study was to test memory performance for words in grapheme–colour synaesthetes and yoked nonsyneasthete controls with a CID-R paradigm to address how synaesthesia affects direct (recognition) and indirect measures of memory (priming and fluency). A secondary aim was to provide a mechanistic account for our results by comparing three formal computational models (SS, MS1, MS2) of direct and indirect measures of memory (Berry et al., 2012) on their ability to account for the behavioural data and to further our understanding on the cognitive processes underlying memory performance in synaesthesia. We found that synaesthetes outperformed controls on memory for word recognition. Numerically, identification times were generally shorter in the synaesthesia sample relative to the control sample. With respect to indirect measures of memory, both groups showed significant priming and fluency effects. While there was a trend for enhanced fluency in the synaesthete compared with the control sample, priming was numerically at best only marginally enhanced in the synaesthete relative to the control sample. Interestingly, the SS model provided the most parsimonious account of data from both groups.

For the first time, we modelled the memory advantage in synaesthesia to further our understanding on the cognitive processes underlying enhanced memory performance in synaesthesia. Without the modelling, there might be a temptation to look at the performance of synaesthetes and conclude that because they only show significant enhancements in recognition and not priming, this pattern reflects enhancement in direct (recognition), but not indirect (priming), measures of memory, thereby supporting the dual coding account of enhanced memory in synaesthesia. However, considering all available evidence, the pattern of the results is very much in line with the enhanced processing account. Recognition performance is significantly enhanced in the synaesthete sample relative to the control sample. Priming is numerically higher in the synaesthete sample relative to the control sample. Identification times during the study phase are numerically faster in the synaesthete sample relative to the control sample (difference of 60 ms). This is also the case for the testing phase (difference of 61 ms). Moreover, there was a statistical trend for enhanced fluency in the synaesthete sample in comparison to the control sample. The SS model was the preferred model when fit to the data, consistent with an account in which the memory advantage in synaesthesia is based on a single source of information and not selective enhancement in a distinct recognition memory signal. The SS model correctly predicts a range of associations between identification RTs and recognition decisions (e.g., enhanced recognition performance is associated with shorter identification times). Consistent with the empirical data, it also predicts a small fluency (9 ms) and priming advantage (13 ms) for the synaesthete group relative to the control group. Crucially, in both MS models, the parameter estimates of the mean strength of both the recognition and identification signals are greater in the synaesthesia group than the control group (see Table 2)—so, again, the performance enhancement cannot be viewed as a selective enhancement in a signal that uniquely drives recognition. This does not directly rule out a dual coding account of the memory advantage in synaesthesia; however, if dual coding is the cause, then the results of the modelling suggest that the mechanism that gives rise to the advantage it produces is not akin to selective enhancement of a distinct memory signal. Instead, the findings seem most parsimoniously explained in terms of enhanced general processing as the *driving factor*.

Two potential explanations for why indirect measures of memory were only numerically, but not significantly, enhanced warrant further consideration. Firstly, the reliability of indirect memory measures is usually lower than the reliability of direct measures of memory. Thus, differential reliabilities of direct and indirect memory measures may be a possible determinant of dissociations between direct and indirect measures of memory as a function of experimental manipulations (Meier & Perrig, 2000). Crucially, effect sizes for performance differences between synaesthetes and controls in recognition and fluency approached both medium sized effects, while the effect size for priming was corresponding to a very small effect. This is consistent with previous research showing that the memory benefit in synaesthesia corresponds to effect sizes from medium to large (Rothen et al., 2012). More generally, this is in line with the notion that synaesthesia leads to an ordinary but not extraordinary memory advantage (i.e., enhanced, but within the normal range; Rothen & Meier, 2010). Secondly, due to equating for encoding strategy during the study phase, our study is likely to provide a conservative estimate of the memory advantage in synaesthesia relative to earlier memory studies in the field, which did not equate for encoding strategy. Even though the synaesthetes, in comparison to the controls, needed on average 60 ms less time to identify the words of the study phase and 61 ms less time to identify the words in the testing phase, they showed a performance benefit in recognition memory, almost a similar benefit in fluency (in terms of effect size), and a slight numerical advantage in priming. Despite the smaller effects sizes for indirect measures of memory, the reliability of the results is further supported by the fact that our study is based on the currently largest yoked sample of synaesthetes and controls. Participants were individually matched for age, gender, education, first language, and handedness. Moreover, it is also one of the largest laboratory-based samples relative to studies which employed less careful matching procedures.

Enhanced processing and dual coding are the main proposed mechanisms to explain the memory advantage in synaesthesia (cf. Rothen et al., 2012). The dual coding account is limited to explain the memory advantage for material eliciting synaesthetic experiences (Gibson, Radvansky, Johnson, & McNerney, 2012; Gross, Neargarder, Caldwell-Harris, & Cronin-Golomb, 2011; Radvansky et al., 2011; Rothen & Meier, 2010; Yaro & Ward, 2007). By contrast, only the enhanced processing account is also able to explain the memory advantage for stimuli which do not elicit synaesthetic experiences (Rothen & Meier, 2010; Rothen et al., 2012; cf. also Yaro & Ward, 2007). Interestingly, the memory advantage in synaesthesia seems to be at least as high or even higher in visual tests than in verbal tests (Rothen & Meier, 2010). It is unclear why there is not an additive benefit for verbal material where enhanced processing and dual coding mechanisms may coexist. Crucially, our findings suggest that the memory advantage in synaesthesia is *predominantly* based on enhanced processing rather than dual coding even when the stimulus material elicits synaesthetic photisms. Thus, visual stimuli which are more complex than visually presented words may even further benefit from enhanced processing. Therefore, it seems reasonable that the memory advantage in synaesthesia is larger for nonverbal stimuli (e.g., complex visual scenes) in comparison to visually presented verbal material.

Synaesthetes outperforming controls in word recognition is consistent with other studies showing that synaesthesia can affect direct measures of memory (e.g., Radvansky et al., 2011; Rothen & Meier, 2010; J. Ward et al., 2013; Yaro & Ward, 2007). Effects of synaesthesia on fluency and repetition priming extends previous research which showed that synaesthesia can affect other indirect measures of memory in classical conditioning tasks (Meier & Rothen, 2007; Rothen et al., 2010), artificial grammar learning (Rothen et al., 2013a), and implicit associative learning (Bankieris & Aslin, 2016). However, future studies will need to test for the extent and the generalizability of these findings. For instance, memory in synaesthesia maybe enhanced for word lists (e.g., Gross et al., 2011; Yaro & Ward, 2007) and short narrative texts (Rothen & Meier, 2010), but not whole conversations or entire books. Moreover, memory may not be enhanced for all different types of synaesthesia (e.g., Isbilen & Krumhansl, 2016).

It is also noteworthy that the SS account of recognition, priming, and fluency, or a unitary signal-detection model (e.g., Berry et al., 2008a; Berry, Shanks, & Henson, 2008b) are preferable to the MS models in predicting enhanced memory. This extends the predictive power of the SS model which so far has only been applied to normal and impaired memory performance, such as in the case of amnesia and aging (e.g., Berry et al., 2014; E. V. Ward, Berry, & Shanks, 2013a, b).

Summarizing, using word stimuli, we tested a relatively large sample of synaesthetes and carefully yoked controls with a CID-R paradigm and applied computational modelling to provide a mechanistic account of memory performance in synaesthesia. Synaesthetes showed enhanced recognition performance, a trend for enhanced fluency, and numerically enhanced priming. The empirical results were most parsimoniously accounted for by an SS computational model of recognition, priming, and fluency, but not MS models. In line with previous findings, our results are more readily explained by the enhanced processing account of the memory advantage in synaesthesia rather than the dual coding account.

### Open practices statement

The data reported here are available at the Open Science Framework (https://osf.io/nyqbp/ or doi:10.17605/OSF.IO/NYQBP). The study has not been preregistered.

## Appendix

Our modelling results indicate that the SS model should be preferred over the MS1 and MS2 models. One potential concern is that the true model may in fact be the MS1 or MS2 model, but that the SS model is able to provide a better fit to the data (e.g., by AIC or BIC) because it is overly flexible and can mimic these models. To investigate this possibility, we conducted model recovery simulations, basing our procedures on those described by Wagenmakers, Ratcliff, Gomez, and Iverson (2004). For each participant, we sampled (with replacement) from their data set as many old and new test trials as were analyzed for that participant. The SS, MS1, and MS2 models were then fit to the data. Next, the parameter estimates were used to simulate 50 old and 50 new item trials for each participant, producing a parametric bootstrap sample (Wagenmakers et al., 2004). The data were simulated with the constraint that there would be at least one hit and one false alarm per participant (as in the empirical data). The models were then fit to the simulated data and compared in the same manner as in the current study. These steps were repeated 1,000 times. Each repetition can be conceptualized as a simulated experiment in which data are generated from the SS, MS1, and MS2 models and the fit of these models to the data is compared. In previous work, model recovery simulations were also conducted with the SS, MS1, and MS2 models (Berry et al., 2012). Note, however, that the simulations in previous work were based on different experimental designs (e.g., designs with multiple within-subjects conditions, or ratings designs), and used different simulation procedures (e.g., parametric bootstrap samples were not derived for every participant), warranting the current simulations.

Table 3 shows the proportion of simulated experiments in which the SS, MS1, and MS2 models were preferred (i.e., recovered) according to the AIC. The pattern of results was identical to that of the BIC. The first and second rows of Table 3 show that the SS and MS1 models could be successfully recovered—that is, they tended to provide the best AIC when fit to data that they themselves had generated. In contrast, the final row of Table 3 shows that the MS2 model could not be recovered and instead tended to be mimicked by the SS model and also, to a lesser degree, the MS1 model. This means that although we can say with some certainty that the empirical data are extremely unlikely to have been generated by the MS1 model, we cannot rule out the possibility that that the MS2 model generated the data. Nevertheless, given that the SS model is able to account for the data with only a single memory strength signal, rather than multiple correlated signals, this does not alter our conclusion that the SS model provides the more parsimonious account. The MS2 model cannot be recovered here, because it has one more free parameter than the other models (see main text), and the penalty it incurs when the AIC (or BIC) is calculated is greater than the improvement in fit (in terms of log likelihood) that its additional parameter provides.

**Table 3:** Proportion of simulated experiments (out of 1,000) in which the SS, MS1, or MS2 model was recovered by the AIC

		Recovered model					
		Synaesthete group (*N* = 32)			Control group (*N* = 32)		
		SS	MS1	MS2	SS	MS1	MS2
Generating model	SS	0.994	0.006	0.000	0.996	0.004	0.000
	MS1	0.025	0.975	0.000	0.003	0.997	0.000
	MS2	0.866	0.134	0.000	0.617	0.383	0.000

## Footnotes

1 Given the yoked samples, paired comparisons could be reasonably justified. All analyses were repeated using paired comparisons. The results did not differ from the reported unpaired comparisons.
